# Leucine mediates autophagosome-lysosome fusion and improves sperm motility by activating the PI3K/Akt pathway

**DOI:** 10.18632/oncotarget.22910

**Published:** 2017-12-04

**Authors:** Jin Zhang, Xuemei Zhang, Yingjie Liu, Zihao Su, Farman Ullah Dawar, Hong Dan, Yan He, Jian-Fang Gui, Jie Mei

**Affiliations:** ^1^ College of Fisheries, Key Laboratory of Freshwater Animal Breeding, Ministry of Agriculture, Huazhong Agricultural University, Wuhan 430070, China; ^2^ State Key Laboratory of Freshwater Ecology and Biotechnology, Institute of Hydrobiology, Chinese Academy of Sciences, University of the Chinese Academy of Sciences, Wuhan 430072, China

**Keywords:** leucine, sperm motility, PI3K/Akt pathway, autophagosome-lysosome fusion

## Abstract

Amino acid supplementation is an efficient and effective strategy to increase sperm quality. In our research, a comparative study was conducted to screen free amino acids to improve sperm motility, and we found that leucine was the most efficient one. Leucine treatment increases sperm motility depending on the activation of PI3K/Akt signaling pathway, while the chemical inhibitor of PI3K/Akt signal could reduce the amount of pAkt activated by leucine treatment. Moreover, leucine treatment improved the expression of P62 and LC3-II, substantially suppressed the autophagy process in zebrafish testis. *In vitro* studies showed that leucine could reduce the fusion of autophagosome and lysosome that was indicated by the co-localization of EGFP-LC3 and lysosome marker. Two chemical modulators of autophagy, such as LY294002 (the inhibitor of PI3K/Akt signal) and chloroquine were administered to investigate the process of autophagy on zebrafish sperm motility. LY294002 inhibited autophagosome formation to reduced sperm motility, while chloroquine inhibited the fusion of autophagosome and lysosome to improve sperm motility. Our data suggest that short-term treatment with leucine could increase zebrafish sperm motility by affecting the autophagy and inhibiting the fusion of autophagosome and lysosomes, depending on the activation of PI3K/Akt signaling pathway.

## INTRODUCTION

Sperm quality is an important factor for successful fertilization and survival of the progeny, which is usually evaluated by multiple quality indexes. Sperm motility, a basic exponent of sperm quality is a quantitative trait and a critical determinant of fertility. Sperm motility can be improved by different molecules including small peptides and compounds [1, 2]. Studies recognized that amino acid supplementation is an efficient strategy to increase sperm motility and fertilization. Arginine was the main free amino acid in the spermatozoa of *Oncorhynchus mykis*s, *Cyprinus carpio* and *Perca fluviatilis*. In addition, arginine, glutamic acid and methionine were found in the semen of several fish species where sperm motility was improved when spermatozoa were co-incubated with methionine [3, 4]. In mouse, cysteine analogs can increase the fertilization of frozen-thawed sperms [5]. Hence, some essential amino acids are likely to be involved in the sperm motility of teleost fishes.

In the male reproductive organs, many endocrine and paracrine factors participate in the regulation of sperm motility and fertility. The increase level of [Ca2+]_i_ and cyclic AMP (cAMP) in the testes trigger the activation of sperm motility and fertilization [1, 2]. Additionally, calcium has been shown to regulate the PI3K-AKT signaling pathway in the testis [6]. Loss of p110beta, a subunit of the phosphoinositide 3-OH kinase leads to impaired spermatogenesis and defective fertility [7]. Depending upon calcium influx, Progesterone activates the PI3K-AKT pathway and mediates motility and hyper-activation of human spermatozoa [8]. Similarly, membrane progestin receptor-alpha activates PI3k/Akt pathway, which further stimulate sperm hyper-motility in Atlantic croaker [9]. In asthenozoospermic patients, the varying expression of PI3K/AKT and cAMP mediated PKA signaling pathways that phosphorylate flagella proteins and regulate sperm motility [10].

Autophagy plays an important role in regulating early reproductive events, such as gamete maturation and fertilization [11, 12]. Autophagy-related protein 7 (Atg7) is essential for mouse acrosome biogenesis during spermatogenesis, depletion of which results in complete infertility with aberrant acrosome formation [13]. Administration of Chloroquine, a well-known autophagy inhibitor, can increase the sperm motility and fertilization in mammals and fish via activation of PI3K-AKT signaling pathway [14–16]. Until now, it is unknown that whether some essential amino acids are involved in the regulation of zebrafish sperm motility. In this study, we recognized a small amino acid, leucine that was beneficial for improving sperm motility in zebrafish.

## RESULTS

### Amino acids can improve zebrafish sperm motility *in vivo*

Ten essential amino acids were selected to investigate their effect on sperm motility in zebrafish. As shown in Table 1, tryptophan, phenylalanine and leucine could significantly improve sperm motility according to the sperm parameters (average path velocity, VAP; straight line velocity, VSL; curvilinear velocity, VCL) compared with control zebrafish. In contrast, valine and cysteine administration reduced sperm motility. Interestingly, leucine could significantly increase all the parameters (VAP, VSL, VCL) of zebrafish sperm.

**Table 1:** A screen of amino acids that could improve zebrafish sperm motility

NAME	VAP	VSL	VCL	Effect
**control**	53.13±1.50	50.03±2.14	69.86±3.02	-
**threonine**	57.76±8.96	51.23±13.73	65.51±6.81	NO
**tryptophan**	70.24±3.24^**^	65.14±3.72^**^	75.92±3.79	UP
**proline**	56.69±5.54	45.38±2.19^**^	79.83±13.97	NO
**valine**	40.73±3.55^**^	30.79±2.48^**^	60.24±12.48	DOWN
**methionine**	52.82±5.55	45.23±7.36	71.23±11.42	NO
**cysteine**	49.77±7.61	42.99±5.01^**^	59.69±5.73^**^	DOWN
**phenylalanine**	70.24±7.63^**^	65.61±7.43^**^	76.49±5.05	UP
**serine**	62.21±2.04^**^	57.25±4.36	73.72±3.63	NO
**leucine**	74.83±3.23^**^	64.19±5.75^**^	76.37±1.77^**^	UP
**glutamate**	49.77±7.61	42.99±5.01	59.69±5.73^**^	NO

VAP, average path velocity; VSL, straight line velocity; VCL, curvilinear velocity. ^*^P < 0.05, ^**^P < 0.01 and ^***^P < 0.001.

### Leucine treatment increases sperm motility via activation of PI3K/Akt signaling pathway

**Figure 1:**
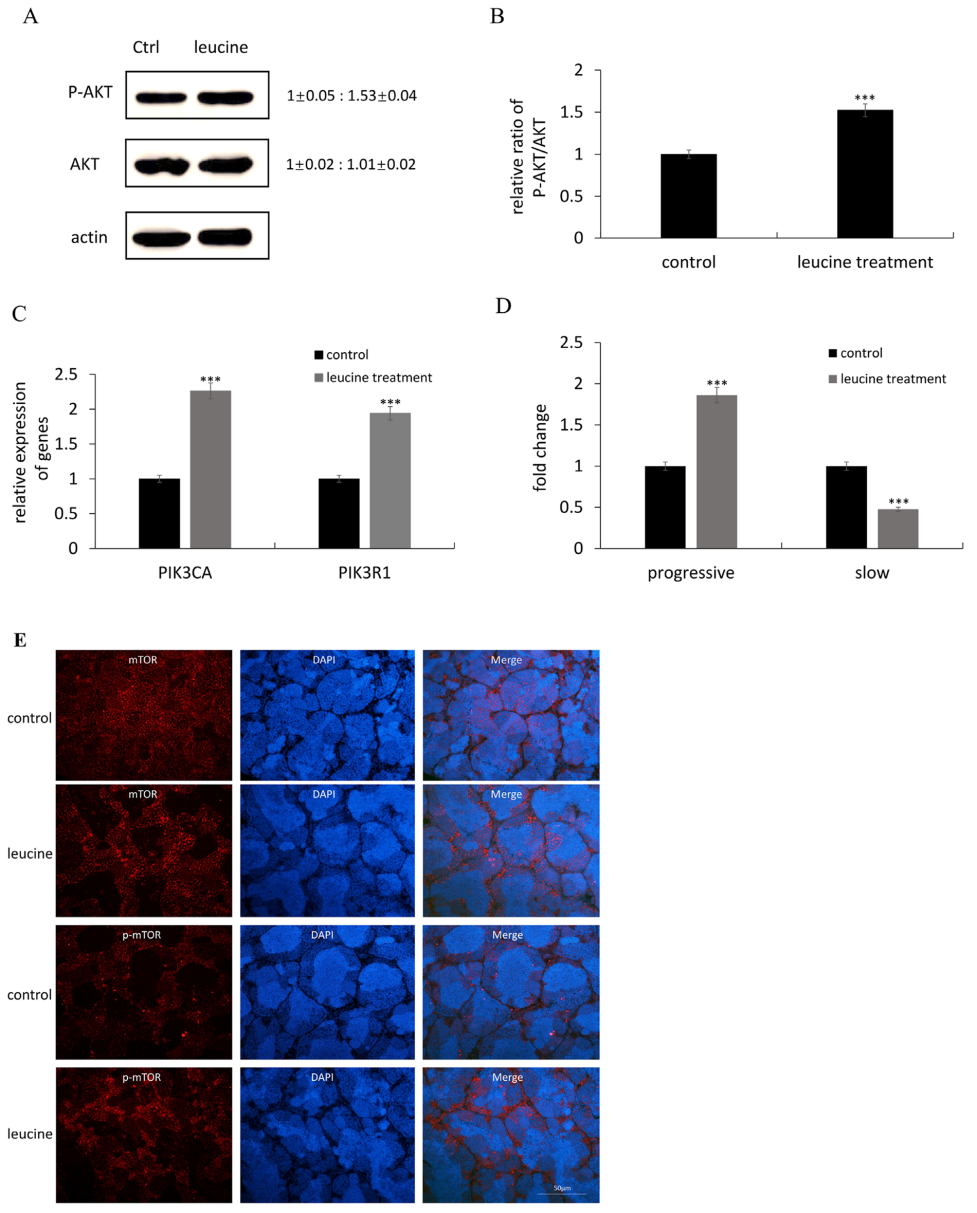
The effects of leucine treatment on zebrafish sperm motility the activation of PI3K/Akt signaling pathway **(A)** Representative immunoblots showed p-Akt and Akt proteins after leucine treatment. Actin was used as the control. Relative protein level was shown from 3 independent experiments. **(B)** Quantification of the ratio of pAkt/Akt from 3 independent experiments. **(C)** The mRNA expression of *PI3KR1* and *PI3KCA* were checked by qRT-PCR. **(D)** Comparisons of semen kinematic parameters between control group and leucine-treated group. Two kinematics parameters including progressive sperm and slow sperm were assessed with the computer-assisted sperm analysis (CASA) system. **(E)** Representative immunofluorescence pictures to detect mTOR and p-mTOR signals in control and leucine-treated zebrafish testes. Scale bar: 50 μm. Bars represent mean ± SEM of more than three separate experiments. ^*^P < 0.05, ^**^P < 0.01 and ^***^P < 0.001.

Leucine was found as one of the efficient amino acids to improve zebrafish sperm motility *in vivo*. Since activation of the PI3K/Akt pathway has been demonstrated to promote sperm motility, we investigated whether leucine treatment could affect this signaling pathway. The protein level of phosphorylated Akt (pAkt) and total Akt (Akt) were checked in the testes of control and leucine-treated zebrafish. As shown in Figure 1A-1B, leucine treatment resulted in a significant increase in the ratio of p-Akt/Akt compared to the control, whereas the level of total Akt was not affected. Moreover, the mRNA levels two PI3K-related genes, *PI3KR1* and *PI3KCA* were induced by leucine treatment (Figure 1C). The percentage of progressive sperm was enhanced after leucine treatment (Figure 1D). Immunocytochemistry was performed to evaluate the expression of mTOR and p-mTOR in zebrafish testis. Compared with the control, the immunostaining signals of both mTOR and p-mTOR were obviously increased by leucine treatment (Figure 1E).

**Figure 2:**
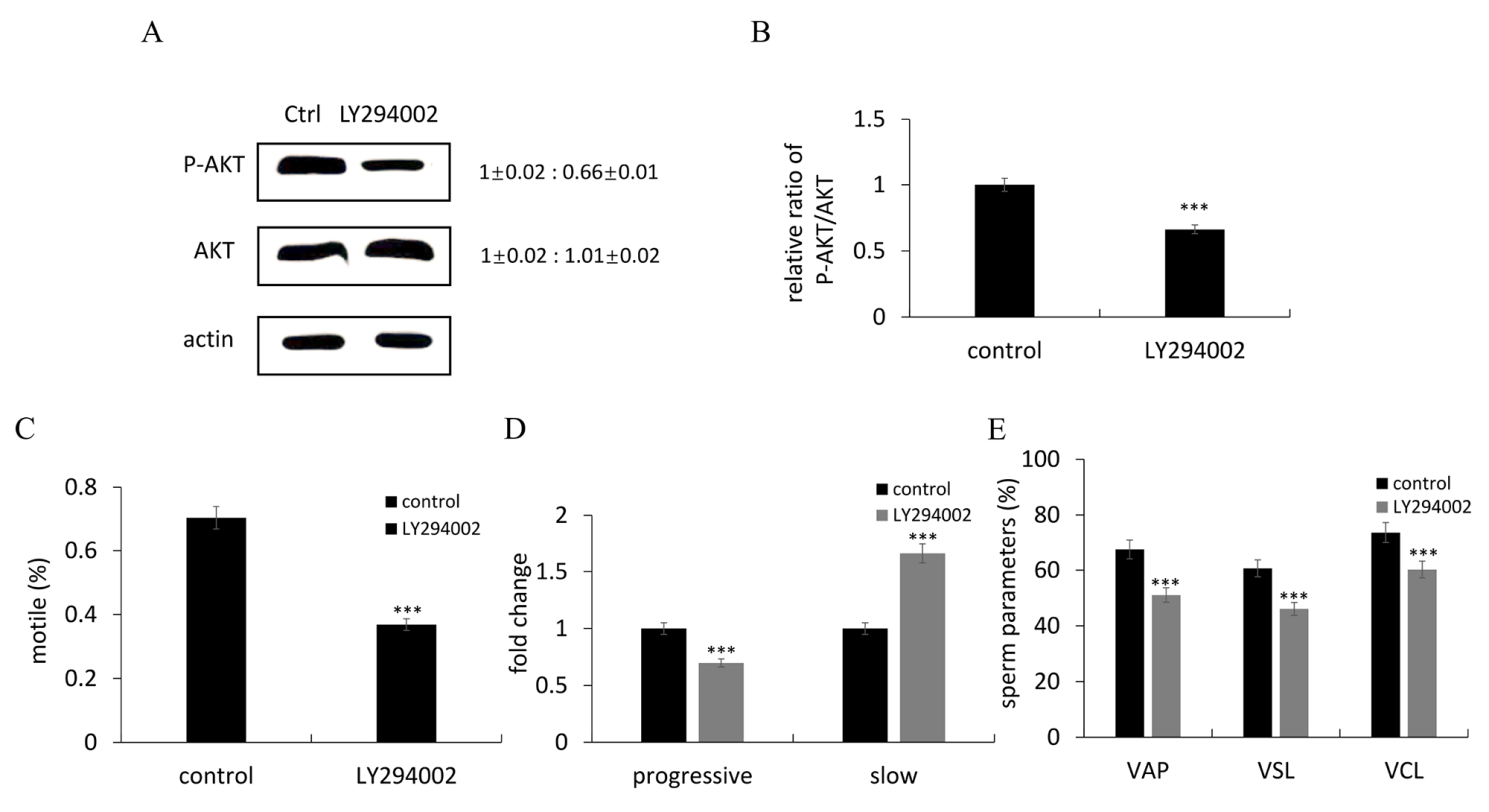
LY294002 suppressed sperm motility through inhibiting the PI3K/Akt signaling pathway **(A)** Representative immunoblots showed p-Akt and Akt proteins after LY294002 treatment. Actin was used as the control. Relative protein level was shown from 3 independent experiments. **(B)** Quantification of the ratio of pAkt/Akt from 3 independent experiments. **(C-E)** Comparisons of semen kinematic parameters between control group and LY294002-treated group. Six kinematics parameters including motile (C), progressive and slow sperm (D), VAP, VSL and VCL (E) were assessed with the computer-assisted sperm analysis (CASA) system. Bars represent mean ± SEM of more than three separate experiments. ^*^P < 0.05, ^**^P < 0.01 and ^***^P < 0.001.

**Figure 3:**
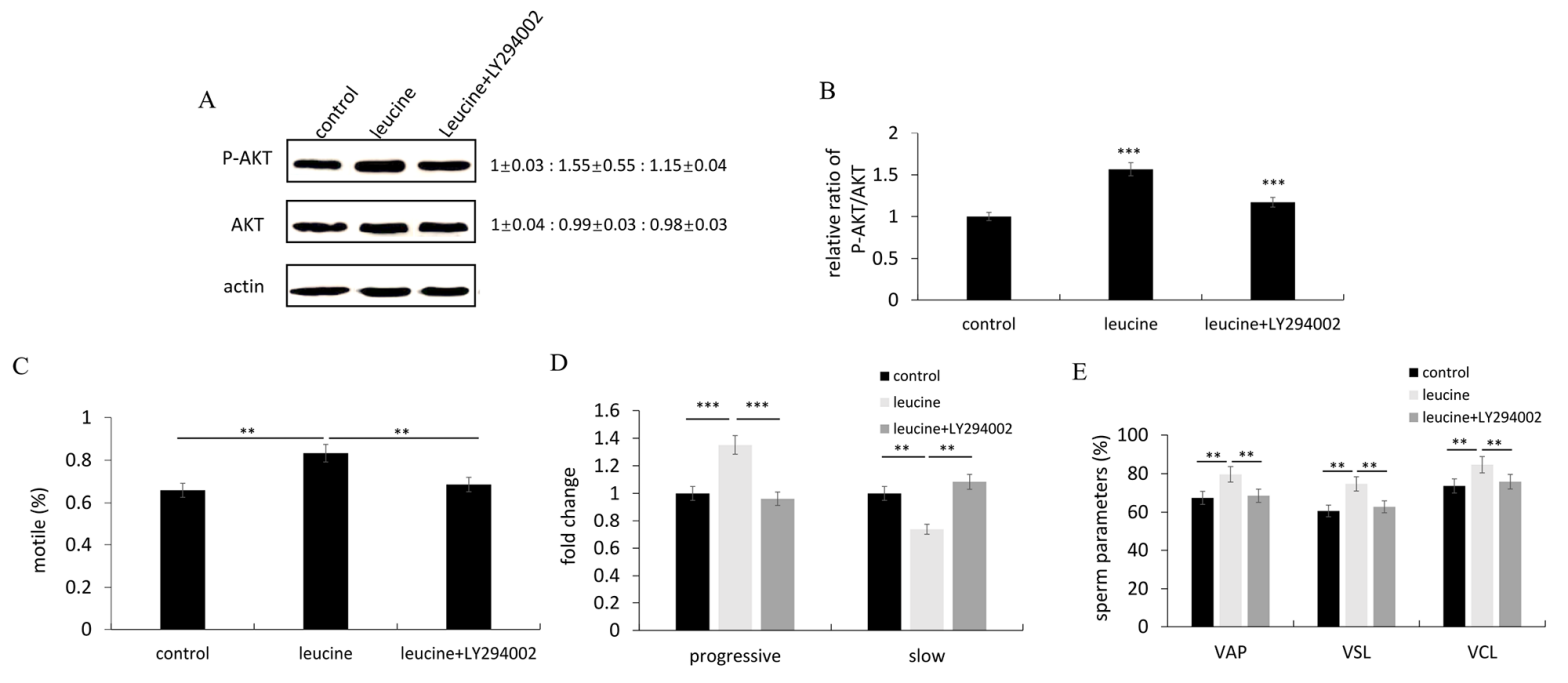
The effects of leucine treatment on zebrafish sperm motility were reduced after inhibiting the PI3K/Akt signaling pathway **(A)** Representative immunoblots showed p-Akt and Akt proteins after LY294002 and Leucine treatment. Actin was used as the control. Relative protein level was shown from 3 independent experiments. **(B)** Quantification of the ratio of pAkt/Akt from 3 independent experiments. **(C-E)** Comparisons of semen kinematic parameters. Six kinematics parameters including motile (C), progressive and slow sperm (D), VAP, VSL and VCL (E) were assessed with the computer-assisted sperm analysis (CASA) system. Bars represent mean ± SEM of more than three separate experiments. ^*^P < 0.05, ^**^P < 0.01 and ^***^P < 0.001.

To investigate the association between leucine and PI3K/AKT pathway in sperm motility, an inhibitor and an agonist of PI3K signal were used. LY294002 treatment significantly inhibited the relative amount of pAkt, and reduced the ratio of p-Akt/Akt compared to the control (Figure 2A-2B). In addition, the values of several kinetic parameters including percentage of motile spermatozoa (Figure 2C), progressive motility (Figure 2D), VAP, VSL and VCL (Figure 2E) were also significantly reduced, respectively. LY294002 effectively reduced the amount of pAkt activated by leucine treatment (Figure 3A-3B). Moreover, the values of several kinetic parameters including percentage of motile spermatozoa (Figure 3C), progressive motility (Figure 3D), VAP, VSL and VCL (Figure 3E) were evaluated, respectively. The values of these kinetic parameters were upregulated after leucine treatment, whereas the effects of leucine on sperm motility were reduced when PI3K/AKT pathway was inhibited by LY294002 treatment.

**Figure 4:**
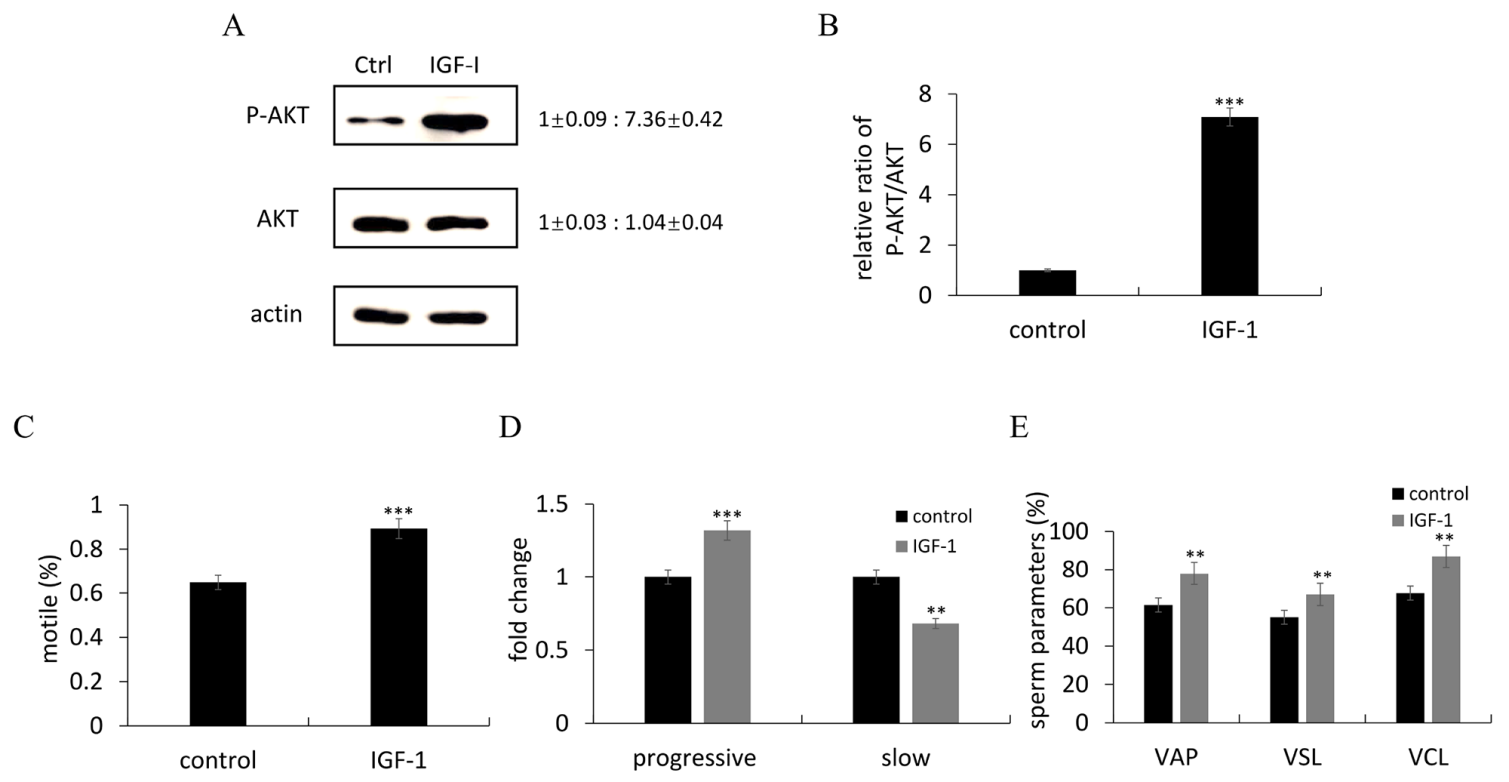
IGF-1 activates the Akt signals and promote sperm motility **(A)** Representative immunoblots showed p-Akt and Akt proteins after IGF-I administration. Actin was used as the control. Relative protein level was shown from 3 independent experiments. **(B)** Quantification of the ratio of pAkt/Akt from 3 independent experiments. **(C-E)** Comparisons of semen kinematic parameters including motile (C), progressive and slow sperm (D), VAP, VSL and VCL (E) that were assessed with the computer-assisted sperm analysis (CASA) system. Bars represent mean ± SEM of more than three separate experiments. ^*^P < 0.05, ^**^P < 0.01 and ^***^P < 0.001.

It is known that Insulin-like growth factor I (IGF- I) is a dominant effector of PI3K/AKT pathway. IGF-1, the agonist of PI3K signal that could significantly increase the ratio of pAkt to total Akt in zebrafish testis (Figure 4A-4B). Moreover, the values of percentage of motile spermatozoa, progressive motility, VAP, VSL and VCL were raised after IGF-1 treatment (Figure 4C-4E).

### Leucine suppresses autophagy by inhibiting the fusion of autophagosome and lysosome

**Figure 5:**
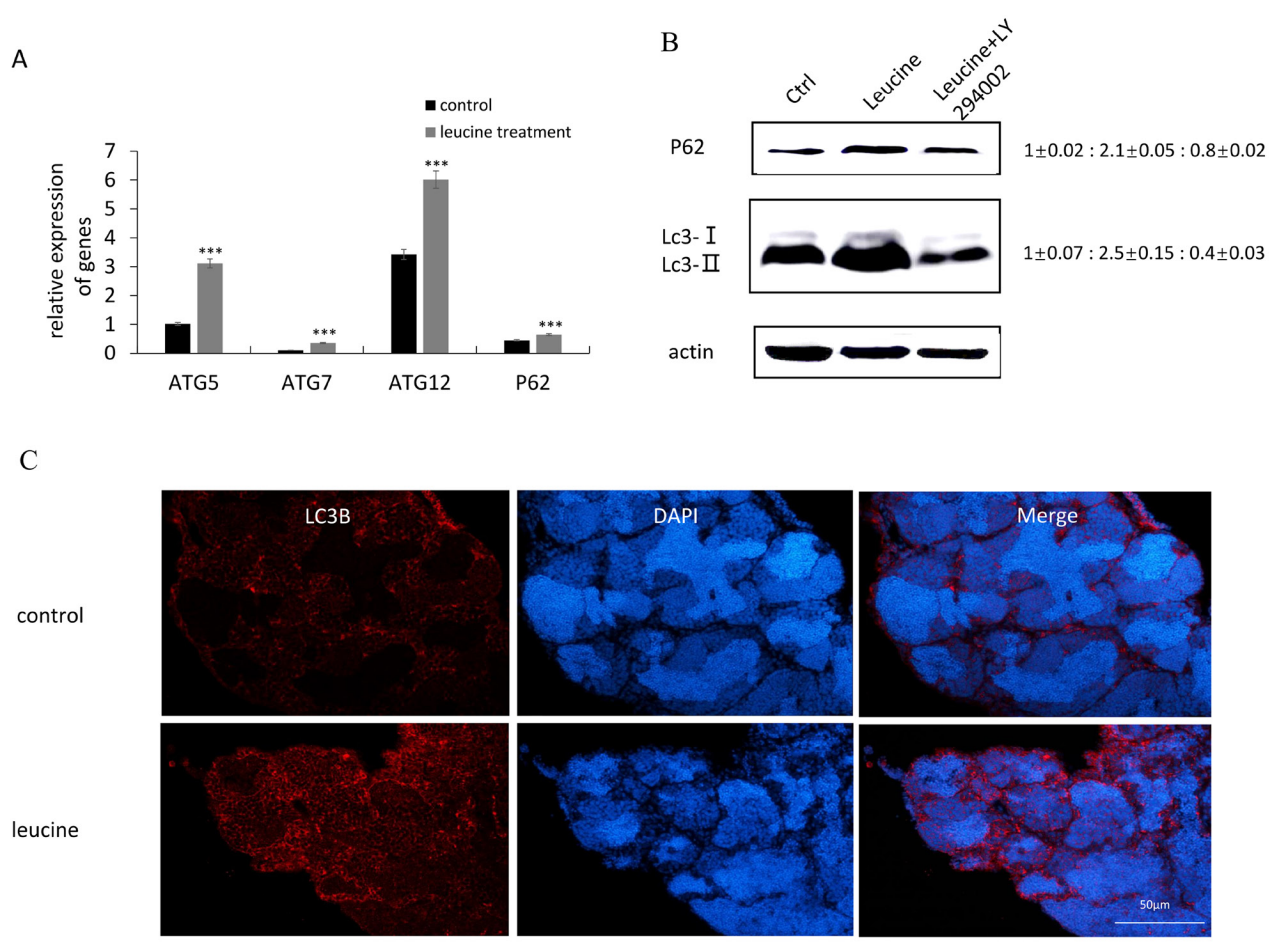
The effects of leucine treatment on autophagy in zebrafish testis **(A)** The mRNA expression of autophagy related genes *atg5*, *atg7*, *atg12* and *p62* were evaluated by qRT-PCR. **(B)** Representative immunoblots showed P62 and Lc3 proteins after leucine and LY294002 administration. Actin was used as the control. Relative protein levels of P62 and Lc3-II were shown from 3 independent experiments. **(C)** Representative immunofluorescence pictures to detect Lc3 levels in control and leucine-treated zebrafish testes. Scale bar: 50 μm. Bars represent mean ± SEM of more than three separate experiments. ^*^P < 0.05, ^**^P < 0.01 and ^***^P < 0.001.

Autophagy is a cellular catabolic process in response to diverse stresses including nutrient deprivation, relying on the cooperation of autophagosomes and lysosomes. After leucine treatment, we observed the expression of autophagy-related genes in zebrafish testis. The results of qRT-PCR indicated that mRNA level of *atg5*, *atg7*, *atg12* and *p62*/*SQSTMl* was significantly increased in response to leucine treatment (Figure 5A). Moreover, autophagy related gene 8 (LC3) and P62/SQSTMl protein were detected by western-blot. Leucine treatment improved the expression of P62 and LC3, especially LC3-II in zebrafish testis compared with control group. When leucine and LY294002 were co-injected into the zebrafish, LC3 expression was significantly decreased compared with control group (Figure 5B). Immunocytochemistry was performed to evaluate the expression of LC3 in zebrafish testis. Compared with low levels of LC3 in the spermatogonia and spermatocyte, and the lack of LC3 expression in the spermatid, the immunostaining signals of LC3 were significantly induced by leucine treatment in these cell types (Figure 5C).

**Figure 6:**
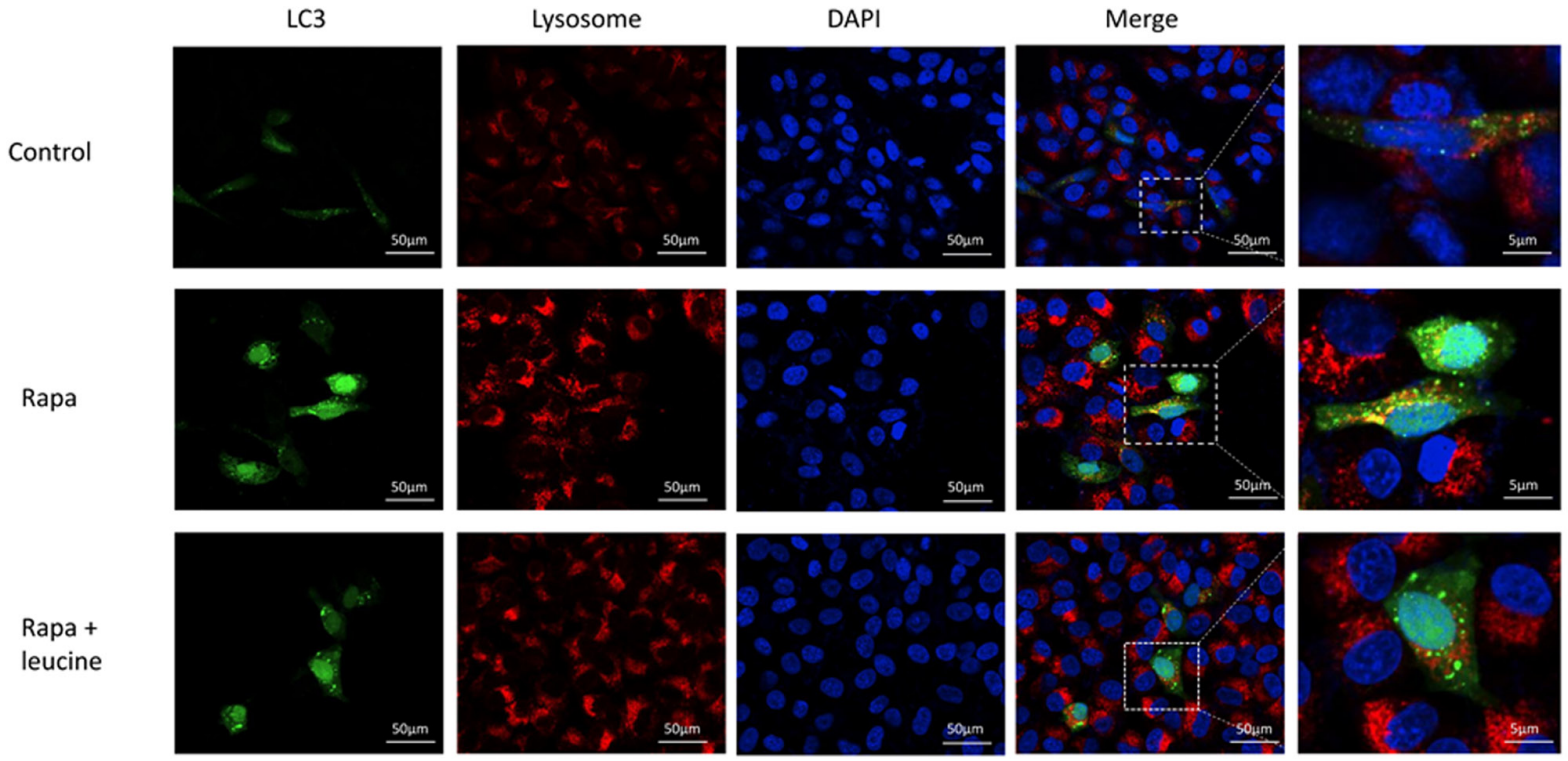
Inhibition of autophagosome-lysosome fusion by leucine in HepG2 cell line Cell imaging was performed to analyze the colocalization of LysoTracker-stained acidified vesicles and GFP-Atg8/Lc3-positive autophagosomes in control and leucine-treated cells. Scale bar: 50 μm and 5 μm. Representative still images of three independent experiments are shown.

The accumulation of LC3-II and p62 protein usually results in an increase of autophagosomes and defects in the fusion of autophagosome and lysosome. To further confirm this statement, EGFP-LC3 vector was transfected into HepG2 cell line. The co-localization of EGFP-LC3 and lysosome marker strongly suggests the autophagosome fusion to lysosome. In the control cells, there was weak EGFP-LC3 signal and very weak co-expression of EGFP-LC3 and lysosome marker (Figure 6). Rapamycin strongly induced the autophagosome formation and the fusion of autophagosome and lysosome (Figure 6). However, when the cells were treated with both leucine and rapamycin, we detected the inhibition of autophagosome and lysosomes fusion that was induced by rapamycin (Figure 6). Our results suggest that leucine could inhibit the fusion of autophagosome and lysosomes.

### Enhancing autophagosome formation and inhibiting the fusion of autophagosome and lysosome is essential for improving sperm motility

**Figure 7:**
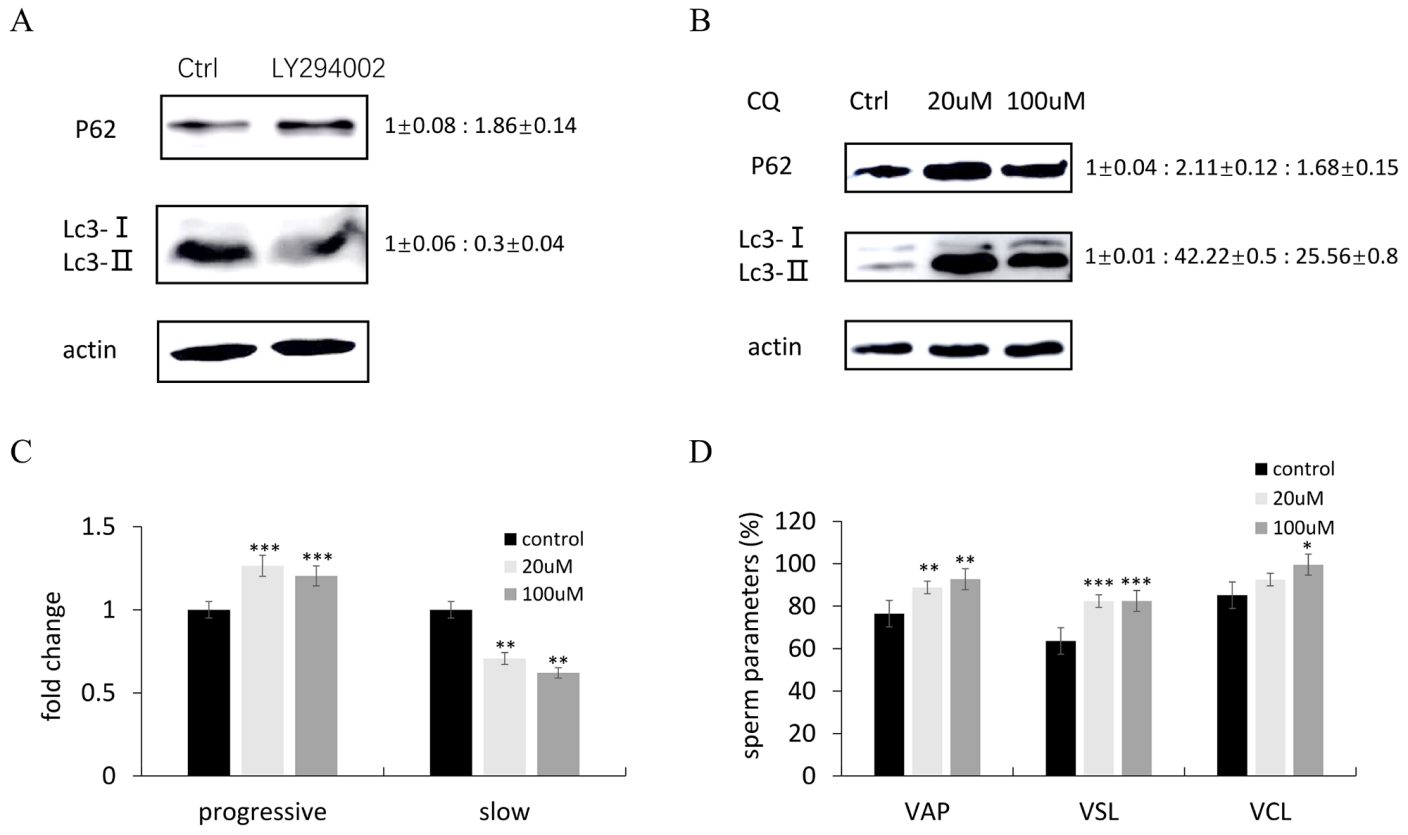
The effects of LY294002 and chloroquine (CQ) on autophagy in zebrafish testis **(A-B)** Representative immunoblots showed P62 and Lc3 proteins after LY294002 (A) or CQ (B) administration. Actin was used as the control. Relative protein levels of P62 and Lc3-II were normalized to the Actin and shown from 3 independent experiments. **(C-D)** Comparisons of semen kinematic parameters between control group and CQ-treated group. Five kinematics parameters including progressive and slow sperm (C), VAP, VSL and VCL (D) were assessed with the computer-assisted sperm analysis (CASA) system. Bars represent mean ± SEM of more than three separate experiments. ^*^P < 0.05, ^**^P < 0.01 and ^***^P < 0.001.

LY294002, the inhibitor of PI3K/AKT pathway has been shown to reduce sperm motility in zebrafish testis (Figure 2). Moreover, LY294002 treatment resulted in upregulation of P62/SQSTMl protein expression and down-regulation of LC3 protein expression (Figure 7A), suggesting that LY294002 could inhibit autophagosome formation. Chloroquine is a common medicine which inhibits the fusion of autophagosome and lysosome via changing lysosomal acidification. Chloroquine-treated zebrafish testes displayed up-regulation of protein expression of both LC3 and P62/SQSTMl at the concentration of 20uM and 100uM (Figure 7B), suggesting that the fusion of autophagosome and lysosomes was inhibited. Meanwhile, the sperm motility parameters were calculated and both treatment groups showed more progressive spermatozoa (Figure 7C), up-regulated parameter values of VAP, VSL and VCL (Figure 7D), compared with the sperm of control zebrafish.

## DISCUSSION

**Figure 8:**
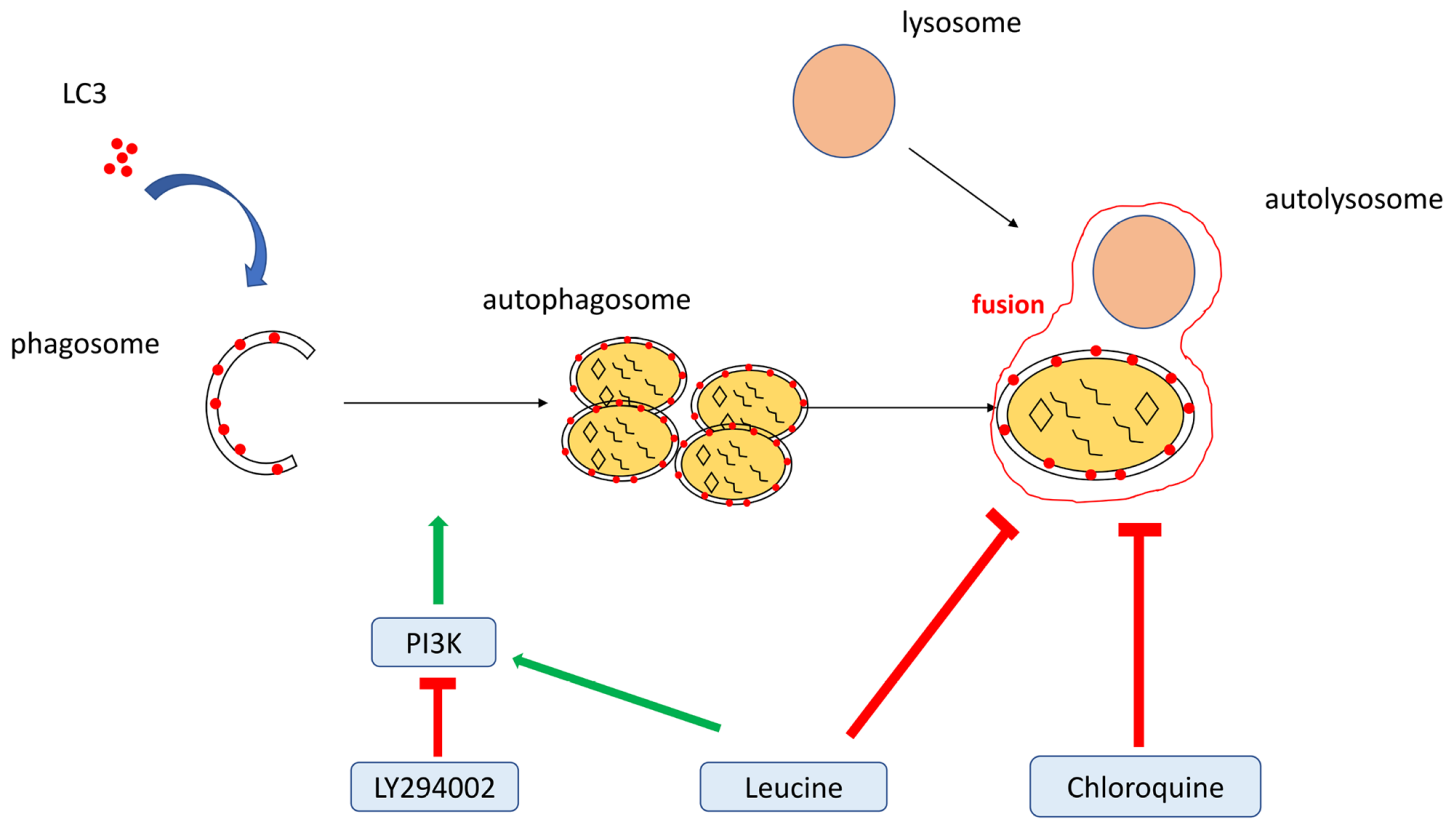
A proposed model for the roles of chemical modulators of autophagy in zebrafish sperm motility LY294002 reduces sperm motility by inhibiting PI3K/Akt pathway and autophagosomes formation. Chloroquine inhibits the fusion of autophagosomes and lysosomes, while leucine actives PI3K/Akt pathway and inhibits the fusion of autophagosomes and lysosomes, subsequently improve sperm motility.

Recently, the damage of male reproductive system and male infertility is increasing due to environmental pollution [17]. Poor semen quality usually caused congenital malformations in the offspring and diseases in the adults. Some small molecules including small peptides and compounds have been shown to significantly improve sperm quality [1, 2]. Autophagy, a natural and physiological process, plays important roles in gamete maturation and fertilization [11, 12]. In addition, administration of chloroquine, a well-known autophagy inhibitor, could increase sperm motility and fertilization rate, with the activation of PI3K-AKT signaling pathway [14–16]. In the current study, we found that leucine can effectively improve sperm motility by promoting autophagosome formation and inhibiting fusion of autophagosome and lysosome, depending on the activation of PI3K/AKT pathway (Figure 8).

PI3K/AKT pathway is involved in the process of male germ cell development. Loss of p110beta, a subunit of the PI3-kinase, results in impaired spermatogenesis and defective fertility [7]. The higher level of PI3K/AKT pathway coincides with a higher degree of testis maturity in YY super-male yellow catfish, compared with the XY male yellow catfish [18, 19]. Inhibition of PI3K/AKT signal by SH5 led to decreased motile spermatozoa with disrupted membrane integrity in stallion sperm [20]. In addition, inhibition of PI3K/AKT signal by wortmannin caused the reduced percentage of motile spermatozoa with the induction of apoptosis [21]. Besides as PI3K inhibitors, wortmannin and LY294002 also work as early-stage autophagy inhibitors through reducing autophagosome formation [22, 23]. PI3K-AKT pathway could be activated by progesterone or membrane progestin receptor-alpha to mediate sperm motility and hyperactivation in human and Atlantic croaker, respectively [8, 9]. Administration of Chloroquine increased the sperm motility and fertilization rate, with the activation of PI3K-AKT signaling pathway in yellow catfish [16]. Leucine treatment has been reported to activate the PI3K/Akt/mTOR signaling pathway [24]. In addition, leucine inhibited myofibrillar proteolysis depending on the PI3K signal, since LY294002, the inhibitor of PI3K could reverse the inhibitory effect [25]. The class IA PI3K subunit genes PIK3CA and PIK3R1 were central signal transducers in the PI3K signaling pathways [26]. The PIK3CA gene encodes the class IA PI3K catalytic subunit p110α, while PIK3R1 gene, encodes the p85α, p55α, and p50α class IA PI3K regulatory subunits. FOXO3a has been reported to trasncriptionally regulate the expression of the PI3K catalytic subunit p110α [27]. In the current study, Leucine treatment increases sperm motility depending on the activation of PI3K/Akt signaling pathway. But how leucine treatment induces PIK3CA and PIK3R1 mRNA expression need further studies. Moreover, leucine rescued the reduction of AKT phosphorylation and sperm motility caused by LY294002 treatment.

Amino acids not only work as substrates to play some physiological roles in various metabolic pathways, but also serve as signaling molecules to regulate signal transduction pathways. For example, mTOR signaling pathway is regulated by several amino acids, particularly leucine [28]. Generally, amino acids have been revealed to stimulate protein synthesis and inhibit autophagy [29–31]. However, the mechanism by which amino acids inhibit autophagy is still obscure. Most of the important amino acids were involved in the regulation of autophagic proteolysis, such as a combination of intracellular leucine with either glutamate or aspartate repressed autophagic proteolysis or lysosomal proteolysis in isolated rat hepatocytes [32, 33]. High concentrations of asparagine could inhibit the fusion of autophagosomes and lysosomes [34]. In L-arginine-induced acute pancreatitis, the fusion of autophagosome with lysosome is disturbed [35]. In diabetic rat, treatment with leucine supplement improved testes morphology and increased the number of live sperm [36]. Our results suggest that leucine administration inhibited the fusion between autophagosomes and lysosomes, thereby improving sperm motility.

Autophagy is a highly conserved degradative pathway that contains two main steps including the formation of autophagosomes and later fusion of autophagosomes with lysosomes and endosomes [37]. Autophagosomes participated in some reproductive events and its activation induced a significant increase in sperm cell survival and motility [38]. When the autophagosome formation was inhibited using LY294002, the sperm motility was suppressed in zebrafish testis (Figure 2C-2E). Chloroquine, an autophagy inhibitor, usually blocks the fusion of autophagosomes with lysosomes, resulting in accumulation of autophagosome both *in vitro* and *in vivo* [39, 40]. Previous studies indicated that short-term administration of chloroquine could improve sperm motility in mammals and fishes [14–16]. Our results indicated that short-term administration of either leucine or chloroquine could increase sperm motility through inhibiting autophagosome-lysosome fusion. Until now, the molecular mechanism underpinning the fusion of autophagosome with lysosome is still not clear. Further studies regarding the correlation and fusion between autophagosome and lysosome for improving sperm quality will contribute to a better understanding of autophagy process in male gamete development and resolve the male reproductive diseases.

## MATERIALS AND METHODS

### Animal and experimental protocols

Sexually mature male zebrafish with similar size were cultured at zebrafish facility in Huazhong Agricultural University. All experimental operations were conducted as the requirement of the institution animal care and use committee of Huazhong Agricultural University. The amino acids were dissolved in ddH_2_O to a concentration of 10μM and 15μl amino acid was intra-peritoneally injected into male individuals once daily for 3 days. RhIGF-I(291-G1-200, R&D) was dissolved in 1×PBS solution to a concentration of 200μg/ml and 20μl IGF-I solution was intra-peritoneally injected into male zebrafish once daily for 3 days. LY294002 (MCE, HY-10108) was dissolved in DMSO and 20μl was injected into healthy male individuals on the enterocoelia, at a dose of 5 mM. The administration of Chloroquine was performed as previously described [16]. The control groups were injected only with 1×PBS solution with the same concentration of DMSO. For each of the independent experiments, three male zebrafish per group were used.

### Evaluation of sperm motility

Sperm samples were collected from control and treated groups after the administration of amino acids, RhIGF-I or AKT inhibitor. Sperm concentration was assessed using a phase-contrast microscope (400×magnification) according to a previous study [41]. In each case, sperm samples were diluted with HBSS solution. Total number of sperm of each individual was calculated by multiplying sperm concentration and sample volume. Spermatozoa motility and kinematic parameters were quantified by CASA II using Animal Motility Software Manual Version 1.4 (Hamilthon-Thorne Research, Beverly, USA) as previously described [16]. The 10×phase-contrast objective was chosen to analyze the spermatozoa, and the movement of sperms in each sample was recorded from at least three randomly selected fields for three times.

### Cell culture and transfection

HepG2 Cells were grown in DMEM (Dulbecco’s modified Eagle’s medium) supplemented with 10% fetal bovine serum. For experimental purposes, HepG2 cells were cultured in 24-well plates and used for detecting the fusion of autophagosome and lysosome by transfecting the vector GFP-LC3. Twelve hours post transfection, cells were treated with rapamycin and leucine. Then, the cells were collected and dissociated by cold RIPA buffer after 24h. Lysosome-tracker Red (Beyotimt, C1046) was used to detect lysosomes.

### Western blot and immunofluorescence analysis

Total proteins of zebrafish testis were extracted using one step animal tissue active protein extraction kit (Sangon, Shanghai, China). Equal amount of protein samples were separated by electrophoresis on 15% SDS-PAGE and transferred onto a nitrocellulose membrane membrane (Merck Millipore). After blocking with 5% non-fat milk in TBST, the membranes were incubated with different primary antibodies, rabbit polyclonal anti-AKT (Cell Signaling Technology, 4060s), anti-P-AKT (Cell Signaling Technology, 4060s), anti-SQSTM1/P62 (MBL,PM045), anti-LC3A/B (Cell Signaling Technology, 4108), mTOR (Servicebio, 20657-1-AP), p-mTOR (Servicebio, ab109268) and anti-β-actin (Cell signaling, 4967S) respectively. The blots were detected with HRP-conjugated secondary antibodies and visualized using ECL Western blotting detection reagents (GE Healthcare). The blots were scanned and quantified with NIH software Image J.

Immunostaining was performed as described [13]. Briefly, the slides were blocked in the blocking solution (5% BSA) for 20 minutes, then the slides were incubated with primary antibody, rabbit anti-LC3B (abcam, ab48394) at 1:100 dilution. Then, the slides were washed three times again in PBST and incubated with secondary antibody. Nuclei were stained with DAPI for 5 minutes in the dark. In the negative control experiments, rabbit normal serum was used as primary antibody.

### Quantitative real-time PCR (qRT-PCR) analysis

Total RNA was isolated from adult zebrafish testes using TRIzol (Invitrogen, USA) and reverse transcribed into cDNA by PrimeScriptTM RT reagent Kit with gDNA Eraser (Stratagene, Takara). The cDNA synthesis and qRT-PCR were accomplished as described using β-actin as the internal control [42]. The primer sequences were designed using Primer Premier 5.0 software (Table 2). All experiments and measurements were performed in triplicate.

**Table 2:** The primers for RT-PCR

Primers	Sequences (5′–3′)	Applications
ATG5-F	TGGAGTATCCCACCGAAGA	Real time PCR
ATG5-R	TGCCGTGAATCATAACCTG	Real time PCR
ATG7-F	ACGGTGATGCTGTTGGTCTG	Real time PCR
ATG7-R	TTTGTCGGTGGATTTGAAGG	Real time PCR
ATG12-F	TCATCTCACGCTTCCTCAA	Real time PCR
ATG12-R	TCACTTCCGAAACACTCAAA	Real time PCR
P62-F	TGGTGCTACTGCCTCTTCTCA	Real time PCR
P62-R	GGGTTACTTTGGTCCGCTTT	Real time PCR
PIK3R1-F	ACATGGCTCTGCAAGATGCT	Real time PCR
PIK3R1-R	GGAGGCATCTCGGACCAAAA	Real time PCR
PIK3CA-F	CGCAATGAGAGGATGAGCGA	Real time PCR
PIK3CA-R	ACGCTGTCACGATGGAACAA	Real time PCR
β-actin-F	TCCCTGTATGCCTCTGGTCGT	Real time PCR
β-actin-R	AAGCTGTAGCCTCTCTCGGTC	Real time PCR

### Statistical analyses

Quantitative data are reported as the mean ± SEM from three independent experiments. Comparisons of different groups were analyzed by student t-test (^*^P < 0.05; ^**^P < 0.01; ^***^P < 0.001).
